# Data acquired by a single object sensor for the detection and quality evaluation of table tennis forehand strokes

**DOI:** 10.1016/j.dib.2020.106504

**Published:** 2020-11-06

**Authors:** Sahar S. Tabrizi, Saeid Pashazadeh, Vajiheh Javani

**Affiliations:** a Department of Computer Engineering, Faculty of Electrical and Computer Engineering, University of Tabriz, Iran; b Department of Information Technology, Faculty of Electrical and Computer Engineering, University of Tabriz, Iran; c Department of Sport Management, Faculty of Physical Education and Sports Sciences, University of Tabriz, Iran

**Keywords:** Table tennis forehand, Inertial measurement unit, Object sensor, Sports activity recognition, Sports activity analysis, Regression, Classification

## Abstract

Shadow-play, is an assistance solution for Table Tennis training, develops novice players' strokes and performing skills, and helps the players' brain to train in terms of the correct positioning and how the proper stroke technique feels. Most currently proposed training assistance systems are rarely used in actual applications, as they are expensive and their setup is complex. Thus, there is a need for a practical and low-cost intelligent system training assistance solution, as well as the possibility of using this solution comfortably to assist players. This paper specifies Forehand shadow play strokes movement and orientation sensory dataset for Table Tennis using a miniaturized low-powered, inexpensive and non- intrusive Inertial Measurement Unit (IMU) BNO055. We mounted the IMU on the center of a standard Table Tennis racket's surface. Eight novices, eight professional players, and three high ranked Table Tennis coaches participated in this research voluntarily. The Racket enabled us to collect players' strokes' time-series data responsively and sensitively. Collected sensory time-series data contains 1570 samples for the Basic, Topspin, and Push Forehand strokes of the players. Besides, all performed strokes were manually labeled and scored by the coaches simultaneously. The sensory dataset contains data from one 9-axis IMU (3- axis Accelerometer, 3- axis gyroscope, and 3- axis magnetometer) and Euler angles (roll, pitch, and yaw angles), mounted on the Racket. Based on the nature of the Forehand movements, the center of the surface was empirically determined to be the appropriate sensor placement in this experiment. We accomplished the collection of all samples under conditions that have been set by the coaches. The authors expect that the collected dataset can be used in a digital shadow-play coaching system to automatically send feedback to novice players when they practice shadow-play Table Tennis strokes individually.

## Specifications Table

**Table:** 

Subject	Engineering
Specific subject area	Human-Computer Interaction, Motion Recognition, Sports Motion Analysis, Object sensor for Forehand strokes recognition of Table Tennis, Motion evaluation, Regression, Classification
Type of data	CSV files relating to sensory data acquired by the IMU sensor CSV files relating to evaluation scores of the Strokes scored manually by the coaches Excel File addressing the level of the players (Professional and Novice)
How data were acquired	A single 9-axis IMU (BNO055) as an Object sensor includes a triaxial accelerometer, gyroscope, and magnetometer and measures Euler angles (roll, pitch, and yaw angles). The coaches labeled the samples manually accordingly depending on the type of Strokes (Basic, Topspin, and Push) and scored them manually based on the experiment evaluation metrics. A USB cable transmits the captured movements and orientations data via a workstation running the data gathering software.
Data format	Raw and processed.
Parameters for data collection	The sensory data were collected from a total of 16 Table Tennis players of mixed gender, aged between 19 and 38 years, who used the IMU mounted Racket. We considered the Basic, Topspin, and Push as the sample type of performed Forehand strokes. The Table Tennis Forehand strokes quality evaluation values were collected from a total of 3 Table Tennis highly educated and ranked coaches who manually scored the performed strokes based on the experiment evaluation metrics
Description of data collection	The players used the IMU mounted racket for samples collection. Based on the experiment's evaluation metrics, the coaches who placed around the Table-Tennis table scored and labeled each subject's performance simultaneously when the strokes are performing. Each player carried out a predetermined stroke performing protocols during data gathering. Steps of capturing samples were as follows: 1) the players started performing strokes, 2) they paused for five seconds under the surveillance of the coaches at the end of each stroke's execution, 3) software stored the captured sensory data into a single CSV file, 4) coaches manually labeled and scored the players' performance.
Data source location	Sports Complex, Gymnasium, and Table Tennis Hall of the University of Tabriz, Tabriz, IRAN.
Data accessibility	Repository name: Mendeley Data Data identification number: doi: 10.17632/b7bc9y232m.3 Direct URL to data: http://dx.doi.org/10.17632/b7bc9y232m.3
Related research article	S.S. Tabrizi, S. Pashazadeh, V. Javani, Comparative Study of Table Tennis Forehand Strokes Classification Using Deep Learning and SVM, J. IEEE Sensors 2020, 20(22), 13552- 13561. https://doi.org/10.1109/JSEN.2020.3005443

## Value of the Data

• Data are useful to validate the use of Object sensors in motion analysis and evaluation of Table Tennis strokes;
• Application developers and researchers in the Machine Learning field with accompanying researcher or professionals in the field of Sport Management can benefit from these sensory data to propose a new shadow-play coaching system based on a single Object sensor; this system may overcome the limitation of the visual sensing modalities;
• Researchers can use the collected data as a benchmark for performance evaluations of different Machine learning algorithms (Deep and shallow) for the classification and regression to recognize the type of strokes and to estimate the performance of players [1];
• The researchers in the sport management field can use the single IMU sensor data of the correct racket motion models of forehand stroke as a benchmark for evaluating the various type of Forehand stroke racket motion;
• By further examining the collected dataset, researchers can observe multiple patterns of the data and identify connections between the data patterns and the level of the players (professional or novel).
• The cooperation of the athletes and trainers in the design and data collection phases of the current research can overcome the challenge mentioned in the recent Rajšp and Fister study [2].

## 1 Data Description

**Fig. 1:**
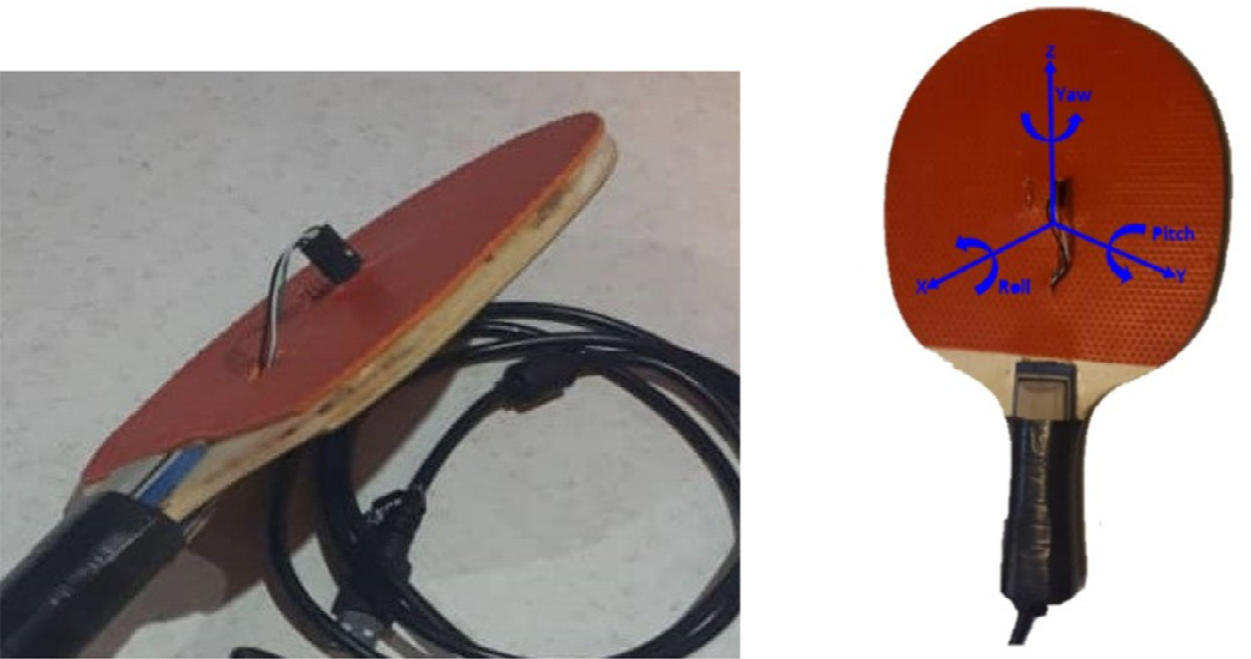
The developed Racket and the position of the IMU sensor on the Racket.

The proposed dataset contains sensory and manually adjusted data captured during the research protocol. It was conducted to validate object sensors measurements in motion detection and quality evaluation of Table Tennis Forehand strokes. The developed Racket used during the data acquisition phase is as shown in Fig. 1. The dataset includes two folders, namely Raw and Processed. Raw folder contains three subfolders, namely "Sensory-data", "Samples-Scores", and "Players-Level". The name of the first subfolder indicates the sensory data that were collected. Each sensory sample is in a Comma Spread Value (CSV) file format (ID.CSV) where ID: 1, 2, ..., 1728. The value of the ID indicates the number of samples. Players-Level's CSV file addresses the relation between ID numbers and the level of the players (Professional or Novice).

Every single ID.CSV file contains the raw values of the racket movements and orientation released from the mounted IMU. Each *ID.csv* file is structured in 14 columns with various rows. Table 1 depicts the file structure and the collected samples' value definitions. The Sample-Scores folder contains a CSV file that shows the average of three scores which was assigned by the coaches based on the experiment's evaluation metrics. The value of the scores represents the quality of the performance. The Scores.CSV file was structured in six columns with 1728 rows. Table 2 depicts the file structure and the collected samples' value definitions. The Players-Level folder contains an XLSX (Excel) file, which shows the relation between the ID of the performed strokes and the ID of the players.

**Table 1:** ID.csv file structure and its values definitions.

# Column	Column Title	Column value	Column definition	Unit
1	#	Time series (s)	acquisition time (sampling frequency=100 Hz)	Digit
2	Time/Date	-	Extra information about the date and time of the data acquisition	String
3	Roll	Euler Angle	Rotation on the *x*-axis	degree
4	Pitch	Euler Angle	Rotation on the *y*-axis	degree
5	Yaw	Euler Angle	Rotation on the *z*-axis	degree
6	Acc(x)	Accelerometer X	acceleration on the *x*-axis of the accelerometer	mg
7	Acc(y)	Accelerometer Y	acceleration on the *y*-axis of the accelerometer	mg
8	Acc(z)	Accelerometer Z	acceleration on the *z*-axis of the accelerometer	mg
9	Gyro(X)	Gyroscope X	angular velocity on the *x*-axis of the gyroscope	Dps
10	Gyro(Y)	Gyroscope Y	angular velocity on the *y*-axis of the gyroscope	Dps
11	Gyro(Z)	Gyroscope Z	angular velocity on the *z*-axis of the gyroscope	Dps
12	Mag(X)	Magnetometer X	magnitude on the *x*-axis of the magnetometer	µT
13	Mag(Y)	Magnetometer Y	magnitude on the *y*-axis of the magnetometer	µT
14	Mag(Z)	Magnetometer Z	magnitude on the *z*-axis of the magnetometer	µT

**Table 2:** Scores.CSV file structure and its values definitions.

# Column	Column Title	Column definition	Unit
1	The ID	The ID of the sample (1–1728)	Digit
2	Criteria #1	Converging and diverging angle of the Racket gripping during the performing (0–100)	%
3	Criteria #2	Forward swing (0–100)	%
4	Criteria #3	Follow-through (0–100)	%
5	Criteria #4	Appropriate speed of the racket movement (0–100)	%
6	Criteria #5	General quality (0–100)	%

The Processed named folder consists of five subfolders, namely "Basic", "Topspin", "Push", "Clean-Players-Level" and "Clean-Sample-Scores". The name of the first three subfolders indicates the label of strokes and contains the captured strokes samples corresponding to the title of the strokes. Like the Raw folder, the other two subfolders are about the level of the players and the quality score of the stroke, respectively. We created the Processed folder contents after completion of the pre-processed phase. The pre-processing stage includes two main sections: 1) eliminate incomplete samples 2) splitting the time series data into fixed and equal segmentation [3].

A Eliminating incomplete samples

We collected 1,728 samples of the participants' Forehand shadow-play strokes. However, 158 of the samples were excluded from this research because the sensor did not capture the signals completely. Thus, 1570 samples are used as raw time-series signals in this paper. Therefore, we excluded the records of incomplete samples from the "Players-Level.csv" and "Scores.csv" files.

B The time-series signals segmentation

**Fig. 2:**
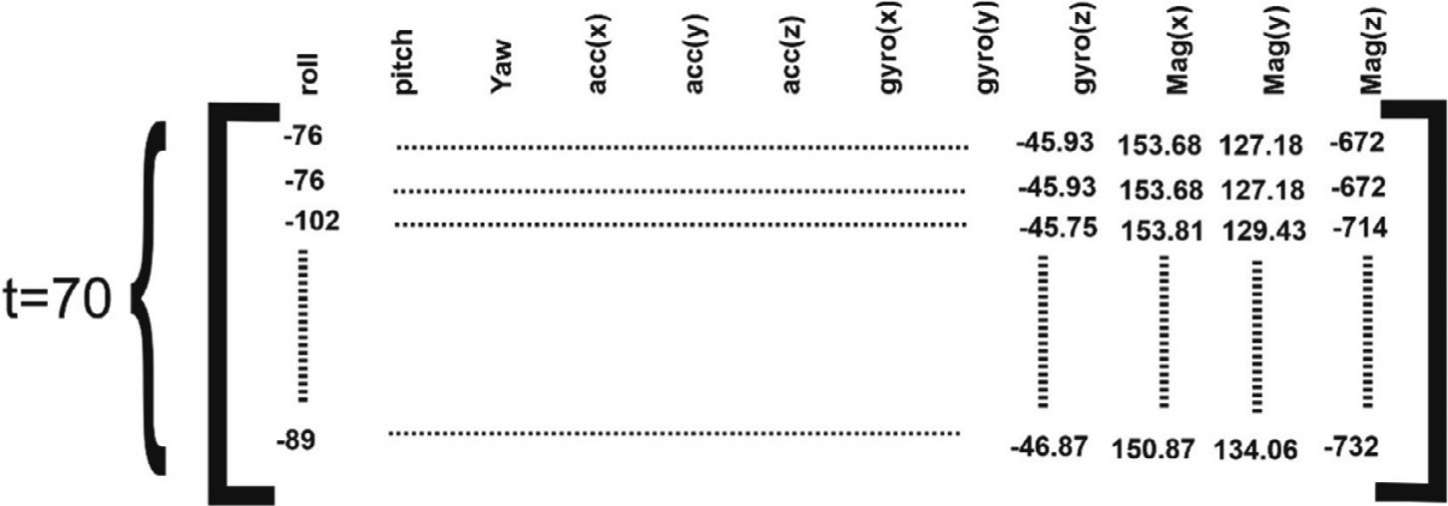
Schema of ID.csv files content.

All acquired signals were passed through the Kalman Filter, and the values of the Euler angles were computed by fusing the sensory data of the IMU. Generally, the primary step in motion recognition tasks based on time series signals involves splitting signals into a fixed and equal length of segmentation by utilizing the sliding window technique [4]. In our paper, we are dealing with quasi-periodic IMUs' signals, where the type of Forehand stroke determines the size and form of the signals. These signals contain crucial data about the corresponding kind of Forehands and play the features role. Based on the coaching approach of the developed system and data acquisition protocols of the research, the collected time-series signals were segmented automatically by players paused for five seconds between two strokes. However, the segmented signals do not have the same form and are not equal in length. In this regard, instead of applying the sliding window technique, the Zero padding technique was executed to make all the segmented time-series signals similar in size (70 signals samples). Zero Paddings is one of the standard data padding techniques used to extend the length of a predetermined segment of data with zero (12 × 70). Fig. 2 shows the storage schema of CSV files.

C The Experiment participants

The data set has been collected by the members of three groups: 1) Professional Table Tennis players 2) Novice Table Tennis Players, and 3) Table Tennis professional Coaches. A group of 16 participants comprising players of both genders participated in the creation of the self-collected dataset. The participants constituted two groups, namely Novice and Professional.

  1 Professional Players

The professional players' group consisted of eight professional players who are experts in Table Tennis and have a national ranking. I.R. Iran Table Tennis Federation (TTF) in East Azerbaijan introduced these players as experienced and highly ranked. Moreover, most of the players also have national and international coaching certificates. All professional players performed Forehand strokes at least 60 times in the limited conditions.

  2 Novice Players

The novice players group consisted of eight voluntary male/female first-year physical education and sport science undergraduate students attending the University of Tabriz Physical Education and Sport Science faculty during the fall semester of the 2019-2020 academic year. The students taking the Table Tennis course for the first time had reached the 4th session of the course. They had already learned about standard positioning and how to grip the Racket. Each novice player performed Forehand strokes at least 30 times in the given situation. It was tried to equalize the gender of the dataset in the selection of the volunteer players (eight females and eight males). Weights and heights' attributes of the players do not have a severe effect on the research's validation. Thus, we did not consider this biometric information.

  3 Coach group

Three high-level ranked coaches who were introduced by I.R. Iran TTF acted as supervisors. They scored and labeled each performed stroke individually. Besides, the coaches contributed to the supervision of the data collection phase as well. The coach supervised each player's stroke performing speed, positioning, the racket gripping style and direction, and the posture of the Forehand strokes.

As seen in Table 3, we captured the movement and orientation of the time series sensory data of Forehand strokes during eight training sessions. Based on the professional group members' availabilities, data were captured in three sessions in various days for this group. We captured shadow play strokes of both groups during the fall semester of the 2019–2020 academic year. This data gathering was accomplished during five sessions of Table Tennis Forehand's training course. 1080 samples were captured for the professional group, and 648 samples were collected from 8 novice players. Overall, 1728 samples were collected during eight training sessions where 788 of the samples are allocated for Basic Forehand, 422 of it are Topspin Forehand strokes, and 518 of the collected samples are Push samples. Table 3 represents the statistical specifications of collected raw samples by detail.

**Table 3:** The statistical specifications of collected raw samples*.

			# Forehand strokes					
Participant	#Participants	Total Samples	Basic	Topspin	Push	Gender	Average Age	Duration
Professional Players	P01	96	16	16	64	F	20–38	3 days
	P02	134	84	0	50	F		
	P03	60	20	20	20	M		
	P04	151	50	50	51	F		
	P05	220	75	75	70	M		
	P06	89	39	25	25	M		
	P07	150	70	10	70	M		
	P08	180	70	90	20	F		
Novice players	N01	100	50	25	25	F	19–22	5 days
	N02	138	43	42	53	F		
	N03	100	50	25	25	F		
	N04	100	50	25	25	M		
	N05	80	50	19	11	F		
	N06	30	21	0	9	M		
	N07	50	50	0	0	M		
	N08	50	50	0	0	M		
Total	16	1728	788	422	518	–	–	8 days

⁎ The coach as a supervisor controlled all collected data in the limited conditions

## 2 Experimental Design, Materials and Methods

A The Experiment Hardware Setup

As seen in Fig. 1, in the current research, We mounted a single IMU (BNOo55) in the center of the Racket. The measured sensory data were transmitted via the USB cable to a workstation. The main reason for choosing this interface is to prevent data from missing. We set the IMU sampling frequency to 100HZ. The data collection software synchronized and recorded the sensory data measured by the IMU. However, the IMU is a factory-calibrated product. The sensor calibration was controlled before sample collecting and during the phase frequently. Players manually headed the direction gripping the Racket (IMU side of the Racket always on top) to obtain the same attitude of the sensor. In this configuration, BNOo55 measured racket movements and orientation in the same direction. General specification of the IMU is presented in Table 4 [5].

B The Experiment Data collection protocols

**Table 4:** General specification of the IMU.

Name	BNO055
size	LGA package 28 pins, Footprint 3.8 mm × 5.2 mm × height 1.13 mm
Digital interface	HID-I2C (windows 8 compatible), 1^2^C, UART V_DDIO_ voltage rate: 1.7–.6 V
Accelerometer features	Programmable functionality: Acceleration ranges: ±2g/±4g/±8g/±16g Low-pass filter bandwidths 1 kHz-<8 Hz
Gyroscope features	Programmable functionality: Ranges switchable from: ±125 Dps to ±2000 Dps Low-pass filter bandwidths 523–12 Hz
Magnetometer features	Flexible functionality: Magnetic field range typical ±1300µT (x-, y-axis); ±2500 µT(z-axis) Magnetic field resolution of ˜ 0.3µT

All participants performed Forehand strokes at least 81 times. Players waited five seconds between each repetition. By utilizing the developed Racket, each captured sample contains 12 different values, where each value represents one of the tri-axis of the IMUs' sensors data separately. Discrete-time series data were collected from participants while they were performing a set of Forehands, namely Basic Forehand, Topspin Forehand, and Push Forehand. All data were collected in the I.R. Iran TTF approved sports hall. The two limiting conditions of the research are, 1) the limit on the speed of the strokes and, 2) the prior need for Forehands training (the positioning, the accurate way to grip the Racket, and posture of the Forehand strokes). The waiting times were the only interruptions during the data collection phase. Players performed the strokes using their skills under the coaches' supervision. Table Tennis racket's movements and orientations while performing any type of stroke are complicated, mainly when focusing on Forehand techniques.

**Fig. 3:**
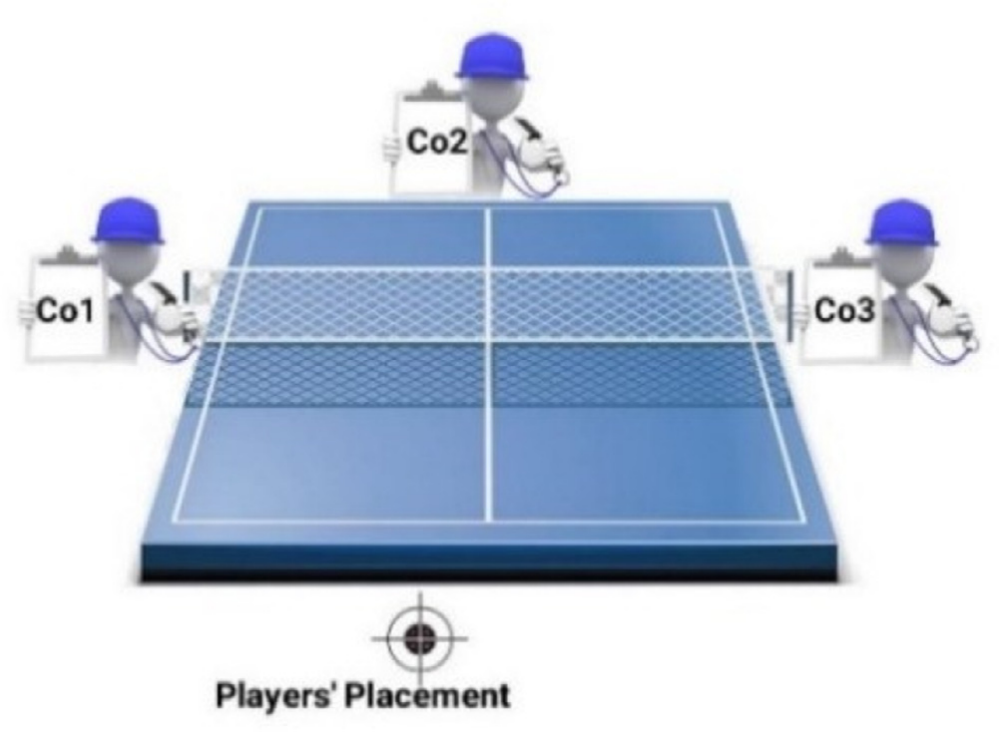
The coaches' placement around the table.

In the case of Forehand strokes, evaluation variables may represent a comparatively high risk of bias. Thus, to avoid personal errors of judgment and obtain approximately more accurate evaluations, three coaches' points of view (Co1, Co2, Co3) were used in this paper. The coaches were placed around the Table Tennis table to observe each player's performance and the assigned scores of performed stroke qualities separately. The places were determined based on the coaches' best-observing conditions and eliminating blind spots associated with the players' arm. Fig. 3 depicts the coaches' placements around the table.

## 3 The Experiment Synchronization, Labeling, and Scoring

A Data collection software

The BNO055 Sensor fusion software (BSX) is used for the recording of the fusion measurements from a 3- axis accelerometer, a 3-axis gyroscope, and a 3-axis magnetometer sensor. Moreover, the software provides the orientation measurements, so-called Euler angles as well.

B Labeling samples

What is more, beyond supervising the initial condition and pause time between two shadow-play executions, the coaches assigned the label of the strokes to every performed sample. The initial state for Forehands training is the positioning, the accurate racket direction (IMU side on top), and the way of gripping the Racket and posture of the Forehand strokes. The coaches noted all collected samples as one of the Forehand strokes type, namely Basic Forehand (B), Topspin (T), and Push (P).

C Scoring samples

Five primary evaluation and scoring Forehand training metrics were adapted among the standard Table Tennis training parameters [6,7] in this paper based on the nature of the shadow-play gentle racket movement and its ball-free training feature. The assessment and scoring criteria for Table Tennis Forehand training in this research is Converging and diverging angle of the Racket gripping during the performing (C1), forward swing (C2), follow-through (C3), appropriate speed of the racket movement (C4), and the performed stroke general quality (C5).

The placed coach group members around the table (see Fig. 3) scored the players' strokes manually. They individually assigned a score (0-100 percentage) to the quality of each performed stroke of each player based on the research evaluating and scoring criterion. At the end of the scoring process, three separate scores lists were yielded.

**Fig. 4:**
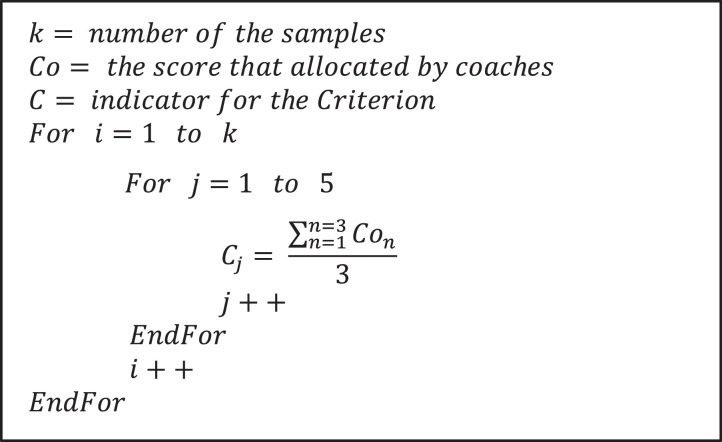
Pseudocode of the process of the scoring strokes.

We used the method of performance scoring of some sports such as Gymnastic and Ice-skating. The following approach was used for each criterion. We used the average of the coaches' scores for each stroke of an individual player. Fig. 4 shows the pseudocode of creating the final score of the strokes.

The practical and close cooperation among all the paper parties (professional players, trainers, and the researchers) in the design and data collection phases of the current research overcomes the challenge mentioned in the recent Rajšp and Fister study [2].

## Ethics Statement

The I.R. Iran TTF in Tabriz Ethics Committee approved the current paper, and all participants delivered their written agreement in advance.

## Declaration of Competing Interest

The authors declare that they have no known competing financial interests or personal relationships that could have appeared to influence the work reported in this paper.

## Appendix Supplementary Materials

Image, application 1

Image, application 2
